# Pathways of ion–molecular interactions of nucleogenic phenyl cations with the nucleophilic centers of picolines

**DOI:** 10.1186/2191-2858-2-14

**Published:** 2012-04-13

**Authors:** Nadezhda E Shchepina, Viktor V Avrorin, Gennady A Badun, Nikolay A Bumagin, Scott B Lewis, Sergey N Shurov

**Affiliations:** 1 Laboratory of Radiochemistry, Natural Sciences Institute of Perm State University, 4 Genkel St, Perm 614990, Russia; 2 Chemistry Department, St-Petersburg State University, 26 Universitetsky Pr, St-Petersburg, Petrodvorets 198504, Russia; 3 Radiochemistry Department, M. V. Lomonosov Moscow State University, Leninskie Gory, Moscow 119992, Russia; 4 Department of Organic Chemistry, M. V. Lomonosov Moscow State University, Leninskie Gory, Moscow 119992, Russia; 5 Department of Chemistry, James Madison University, Harrisonburg, VA 22807, USA; 6 Department of Organic Chemistry, Perm State University, 15 Bukirev St, Perm 614990, Russia

**Keywords:** nucleogenic phenyl cations, picoline, tritium-labeled biological markers

## Abstract

**Background:**

The nuclear-chemical method brought unique opportunity for synthesis of unknown and hardly available organic compounds. Presence of tritium labeling allows one-step preparation of radioactive markers for the investigation of chemical and biological processes.

**Methods:**

The ion–molecular reactions of nucleogenic phenyl cations with 4-picoline have been carried out. The phenyl cations were generated by spontaneous tritium β-decay within the tritium-labeled benzene. Both additions to the nitrogen and substitutions about the aromatic ring were able to be studied simultaneously.

**Results:**

Unusual substitutions on both the α- and β-positions of the ring system have been revealed.

**Conclusion:**

By unknown direct phenylation of nitrogen atom tritium-labeled *N*-phenylpicolinium derivatives, perspective biological markers have been synthesized.

## Background

The pyridine ring itself is a part of many natural and synthetically prepared pharmaceuticals [1,2]. In addition, the pyridine moiety plays a significant role in many biological processes [3,4]. Methyl-substituted pyridines, i.e., picolines, are not the exception in the list of important biologically active substances [5]. The observed increase of biological activity with the introduction of methyl group into the heterocyclic pyridine ring inspires synthetic chemists to find new methods for the preparation of derivatives with picoline fragment [6-11], and biologists for the detail investigations of biological mechanisms with the use of tritium markers [12-14]. Elaborated nuclear-chemical method opens the horizons for synthesis of unknown and hardly available organic compounds. Presence of tritium labeling allows one-step preparation of radioactive markers for the investigation of chemical and biological processes.

## Methods

Previously, we have used the elaborated nuclear-chemical method for the detailed investigations of the ion–molecular reactions of pyridine and quinoline derivatives [15-17]. By these investigations it was shown that with the application of nucleogenic phenyl cations (cations generated by the processes of tritium β-decay), unusual direct phenylation of nitrogen atom in pyridine ring might be realized. Since by the classical methods of chemistry *N*-phenylpicolinium derivatives could not be prepared by phenylation reaction [18,19] and simultaneously syntheses of such compounds with the fixed tritium label are hardly available; our special interest was focused on prolongation of nuclear-chemical method on methyl-substituted pyridines and detail investigation of the pathways of ion–molecular interactions of nucleogenic phenyl cations with the 4-picoline nucleophilic centers.

**Scheme 1:**
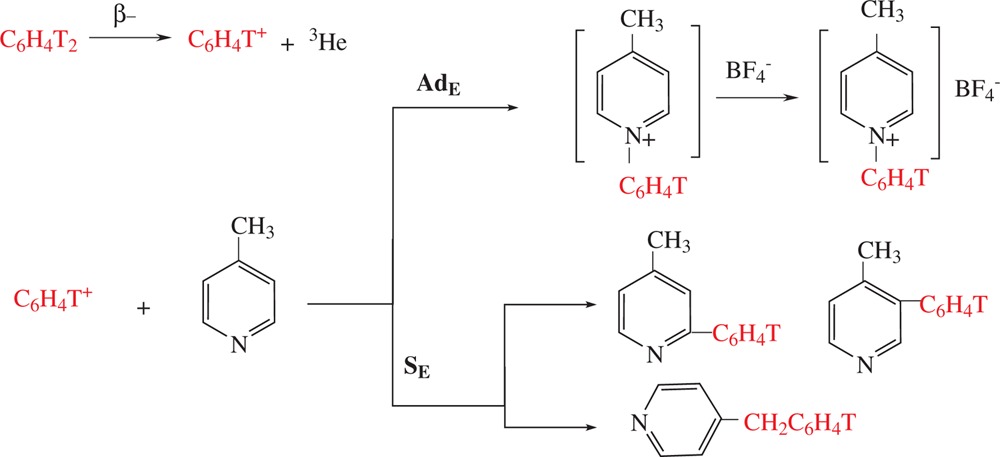
Proposed ion-molecular reactions of nucleogenic phenyl cations with 4-picoline.

Free phenyl cations generated by tritium β-decay in *p-*ditritiated benzene (double labeled benzene is necessary since the second tritium atom is a radioactive tracer) immediately attack the existed nucleophilic centers of the picoline molecule. As it was seen with pyridine, addition to the nitrogen atom to produce *N*-phenylpicolinium salts or substitution on the ring to produce phenyl picolines should be possible. In methylpyridines, also the substitution of hydrogen in methyl group with the formation of benzyl derivative may take place. Projected outcomes for the ion–molecular reactions in the case of 4-picoline are presented in Scheme 1

The ion–molecular reactions of the 4-picoline with the tritium-labeled benzene were carried out in sealed glass ampoules. The ampoules also contained a large excess of a stabilizing salt (KBF_4_). The tritiated benzene used in this study was synthesized from *p*-dibromobenzene by catalytic exchange [15]. After an appropriate accumulation time for the determination of products by radioactivity (approximately 1 month) the reaction mixtures were subjected to TLC analysis. Radio-chromatography of tritium-labeled compounds and comparison with the reference compounds allowed determination of the yields for the products. For identification of the products, in the case of 4-picoline, we have undertaken the synthesis of reference compounds. Methods of synthesis and characterization of these compounds: *N*-phenyl-4-methylpyridinium tetrafluoroborate, 3-phenyl-4-picoline (proposed major substitution isomer), and 2-phenyl-4-picoline are presented in the “Experimental” section.

**Figure 1:**
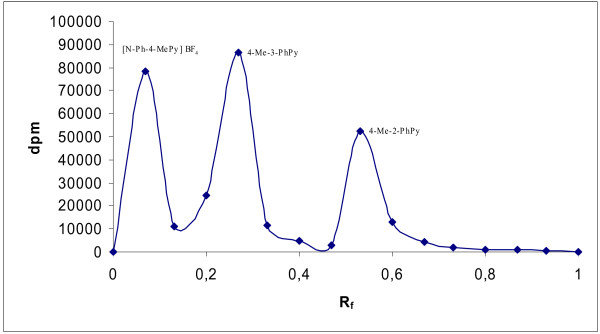
Radioactivity distribution of tritium-labeled products on the chromatographic plates for the reaction of nucleogenic phenyl cations with 4-picoline.

A typical radiochromatogram of the obtained tritiated products from the ion–molecular reactions of tritiated benzene with 4-picoline is presented in Figure 1. From the peak shapes and analysis of the chromatographic peaks with the help of the program “Fityk”, it was possible to confirm that each obtained peak describes the chromatographic behavior of only one substance, not a mixture of substances.

Tritium β-radioactivity has been measured by liquid scintillation counter. Relative yields of ion–molecular reaction products were determined as the ratio of the radioactivity of an individual substance toward the sum of all tritium-labeled products.

## Results and discussion

The radiochemical yields of the addition and substitution products from the reactions of the picoline derivative with the phenyl cations are presented in Table 1.

**Table 1:** Radiochemical yields of products from the addition and electrophilic substitution reactions with 4-methylpyridine

**Substrate**	**Yield, % (Ad_E_)**	**Yield, % (S_E_)**		
4-Methylpyridine	25 ± 2.0	Σ 72 ± 1.0		
		*o-*	*m-*	Unidentified products
		22 ± 3.0	41 ± 2.0	9.0 ± 2.0

It was shown that methyl group in pyridine accents the direction of the interactions from the electrophilic addition of phenyl cations with the unshared electron pair of nitrogen atom toward the electrophilic substitution. And that causes the decrease in the yield of pyridinium salt (the yield in the case of unsubstituted pyridine was about 60%). The methyl group on the picoline is known to stabilize azaarene ions by being an electron donor [20,21]. This stabilization of the azaarene ion can make substitution on to the ring a more favorable process than even addition to the nitrogen atom. The data presented in Table 1 bear this out. We have found that 3-substitution by the phenyl cation was the preferential substitution product (41%). The compound 2-phenyl-4-picoline was produced in approximately 20%. These results fall well in line with the results published for the reactions of pyridine with phenyl cations, in which all three substituted isomers were discovered [22].

Explanation of this noncoincidence with the results of classic chemistry may have several explanations. Since nucleogenic phenyl cations are free, steric effects (especially from offending solvent molecules) become unimportant. We have observed this fact in our synthesis of previously unknown tetraphenylammonium compounds [23]. Moreover, during classical electrophilic reaction on heterocyclic system deactivation of pyridine ring takes place due to the protonation of nitrogen atom by strong acids used for these reactions [24]. Such process does not exist in the ion–molecular reactions with the nucleogenic phenyl cations.

Formation of some amount of the unidentified products probably may refer to possibility of the interaction of phenyl cations with the methyl group of the heterocyclic ring.

## Experimental

General experimental for nuclear-chemical synthesis: All ion–molecular reactions were carried out in sealed glass ampoules containing the source of the phenyl cations (tritiated benzene), the nucleophile of interest (4-picoline) and filled with an inorganic salt to serve as a stabilizing anion (KBF_4_). The molar ratio for benzene and substrate was about 1:1000 (1 μL of hexane solution of tritium double-labeled benzene 4 Cu/sm^3^ and 5.3 μL of the picoline). The ampoules were sealed cold and kept at 0–5°C during accumulation time (about 1 month). After this period, the radioactivity of the obtained products is enough for the detection. The ampoules were opened, solvent added (acetone), and the mixture subjected to TLC analysis on glass plates (Reverse phase C18 silica, fluorescent indicator, CH_3_CN). Radioactivity of the sorbent layer was measured using a scintillation spectrometer RackBeta 1215 (LKB Wallac, Finland).

### Synthetic preparation of *p*-ditritiobenzene

The reaction of catalytic substitution of halogen atoms by tritium in a molecule of *p-*dibromobenzene serves as a basis for synthesis of tritium double-labeled benzene: from 5 mg of dibromobenzene, 6.5 μL thriethylamine diluted in 0.5 mL of hexane (addition of triethylamine is necessary for binding of the formed hydrogen bromide) and 5 Cu of gaseous tritium by hydrogenation at room temperature on 5% Pd/BaSO_4_ catalyst during 1 h the solution of tritium double-labeled benzene has been obtained. The chemical purity was not less than 99%. The volume specific activity of the synthesized benzene came to 4 Cu/cm^3^.

### Synthesis of *N*-phenyl-4-picolinium tetrafluoroborate

*N*-phenyl-4-picolinium tetrafluoroborate was synthesized by a two-step method [25]. The first step was the reaction of 2,4-dinitrobromobenzene with 4-picoline resulting in *N*-(2,4-dinitrophenyl)-4-picolinium salt formation. The last one was converted to *N*-phenyl-4-picolinium tetrafluoroborate by reaction with aniline. *N*-phenyl-4-picolinium tetrafluoroborate was obtained as yellow–orange crystals, m.p. 68–70°C.

### General procedure for the synthesis of 2(or 3)-phenyl-4-picoline

A 20-mL Schlenk tube was charged with 2(or 3)-bromo-4-methylpyridine (0.172 g, 1 mmol), phenylboronic acid (0.146 g, 1.2 mmol), K_2_CO_3_ (0.276 g, 2 mmol), dioxane (7 mL), water (3 mL), and PdCl_2_(PPh_3_)_2_ (0.0071 g, 0.01 mmol, 1 mol% of Pd) under argon. The reaction mixture was placed in a pre-heated oil bath at 150°C and stirred under reflux for 1 h. After this time, the reaction mixture was diluted with 20-mL H_2_O and extracted with Et_2_O (2 × 10 mL). The combined organic layers were washed with 5% KOH (2 × 5 mL), H_2_O (2 × 5 mL), brine (2 × 5 mL), dried over Na_2_SO_4_ and filtrated through a pad of silica gel. The solvent was removed by evaporation in vacuum.

#### *2-Phenyl-4-picoline*

A pale yellow oil was obtained. Yield: 0.156 g (92%). ^1^H NMR (acetone-d_6_, 400 MHz): 2.39 (s, 3H), 7.12 (d, *J* = 4.80 Hz, 1H), 7.40 (t, 1H), 7,46 (t, 2H), 7.74 (s, 1H), 8.11 (d, *J* = 8.08 Hz, 2H), 8.50 (d, *J* = 4.80 Hz, 1H); Anal. Calcd for C_12_H_11_N: C, 85.17; H, 6.55; N, 8.28. Found: C, 85.21; H, 6.62; N, 8.17.

#### *3-Phenyl-4-picoline*

A pale yellow oil was obtained. Yield: 0.162 g (96%). ^1^H NMR (acetone-d_6_, 400 MHz): 2.26 (s, 3H), 7.25 (d, *J* = 4.55 Hz, 1H), 7.37 (d, 2H), 7,40 (t, 1H), 7.47 (t, 2H), 8.37 (s, 1H), 8.41 (d, *J* = 4.80 Hz, 1H); Anal. Calcd for C_12_H_11_N: C, 85.17; H, 6.55; N, 8.28. Found: C, 85.26; H, 6.48; N, 8.26.

## Conclusions

With the use of nucleogenic phenyl cations, the direct phenylation of nitrogen atom in 4-methylpyridine followed by the formation of tritium-labeled biological marker, i.e., *N*-phenylpicolinium salt has been realized. Investigations of the pathways of ion–molecular interactions of nucleogenic phenyl cations with the nucleophilic centers of 4-picoline have been revealed the unusual electrophilic substitution on both the α- and β-positions of the heterocyclic ring system.

## Competing interests

The authors declare that they have no competing interests.

## Authors’ contributions

All authors have contributed equally to this wok. All authors read and approved the final manuscript.
